# Mechanical Analysis and Multi-Objective Optimization of Origami-Inspired Tree-Shaped Thin-Walled Structures Under Axial Impacts

**DOI:** 10.3390/biomimetics10100705

**Published:** 2025-10-17

**Authors:** Honghao Zhang, Zilong Meng, Jixiang Zhang, Xinyu Hao, Shangbin Zhang, Niancheng Guo

**Affiliations:** 1 School of Mechanical Engineering, Shandong University, Jinan 250061, China; honghao_zhang@sdu.edu.cn (H.Z.); mengzilong@mail.sdu.edu.cn (Z.M.); zhangjixiang@mail.sdu.edu.cn (J.Z.); haoxinyu214@163.com (X.H.); zzshangbin0396@163.com (S.Z.); 2 Key Laboratory of High Efficiency and Clean Mechanical Manufacture of Ministry of Education, Shandong University, Jinan 250061, China

**Keywords:** origami-inspired tree-shaped, multi-objective optimization, crashworthiness

## Abstract

Rail vehicles, frequently utilized as a heavy-duty, high-speed means of transportation, have been observed to result in substantial casualties and economic losses in the event of accidents. Energy-absorbing structures are critical to achieving passive safety, effectively absorbing and dissipating energy. The present study utilizes numerical simulation to assess the performance of origami-inspired tree-shaped structures (OTSs) under diverse surface configurations. OTSs offer significant advantages in reducing *IPCF* without substantially compromising other performance metrics. This experimental approach is employed to validate the efficacy of a finite element model. A multi-criteria decision-making method integrates MOEA/D-DAE and TOPSIS. This integrated approach is employed to identify optimal structures. The validity of the method was established through a comparison of the predicted results with the outcomes of finite element analysis. The findings demonstrated a 31.2% reduction in *IPCF*, a 3.6% increase in *SEA*, and a 10.4% rise in *ULC*. The optimized *IPCF* is 4.9919 kN, *SEA* is 12.316 kJ/kg. The collective results indicate the efficacy of the method as a tool for analyzing and optimizing energy-absorbing structures.

## 1. Introduction

Railways play a crucial role in transportation, thanks to their strong load-bearing capacity. However, due to the high speed and heavy weight of trains, accidents can result in a large number of casualties and severe injuries, both in terms of the number of people affected and the severity of injuries. In addition, the sudden nature of such accidents makes it extremely difficult to prevent them completely [1,2]. Thin-walled tubes are widely used in energy-absorbing structural designs due to their simple manufacturing process, low cost, excellent load-bearing performance, and high energy absorption efficiency [3,4]. Extensive research indicates that under axial impact loading, thin-walled structures primarily convert kinetic energy generated during collision into internal energy through plastic deformation, alongside elastic deformation, friction energy, light energy, and sound energy, thereby achieving energy dissipation [5]. Different deformation patterns can significantly impact energy absorption performance [6,7].

Numerous studies have demonstrated that biomimetic energy-absorbing structures exhibit superior energy absorption and collision resistance compared to traditional structures [8,9]. When designing biomimetic energy-absorbing structures, designers often draw inspiration from plants thriving in harsh environments or the tentacles of animals frequently engaged in combat and collisions, using these as biomimetic prototypes for research. New biomimetic energy-absorbing mechanisms are then designed based on analyses of similar structures, loads, and functions. Macro-scale structures such as the dactyl clubs of mantis shrimp [10], honeycomb [11], turtle shells [12], bamboo joints [13], deep-sea glass sponges [14,15], lotus leaf stalks [16], and rice straw folds [17] are widely used in structural design because they are easily observable in nature.

In recent years, the integration of 3D printing technology with origami and biomimetic principles has driven breakthroughs in the mechanical properties and functional integration of lightweight structures [18]. Related research has focused on configuration innovation, performance regulation, and engineering applications, yielding a series of achievements. Wang et al. [19] incorporated origami design into the multi-cell thin-walled structure of high-speed trains. By optimizing the twist angle, collapse zone height, and wall thickness, they reduced the initial peak impact force by 29.36% without compromising energy absorption, thereby validating the effectiveness of origami configurations in enhancing train collision safety. Dalaq et al. [20] studied 3D-printed Kresling origami springs, demonstrating that their mechanical behavior can be regulated through pre-deformation height, pre-deformation angle, and aspect ratio, exhibiting diverse characteristics such as linear, quasi-zero stiffness, nonlinear single-stable, and bistable states. Xia et al. [21] designed a 3D-printed double-layer helical honeycomb (DLHH). By introducing a double-layer helical configuration within the unit cell, the DLHHs reveal a 45% enhancement in the strength and a 200% enhancement in the *SEA*. Dalaq et al. [22] developed a 3D-printed honeycomb pad material based on the Kresling pattern, which demonstrated 70% energy absorption efficiency and 94% energy dissipation efficiency, and exhibited full shape recovery capability, offering a new solution for reusable impact protection materials. Ma et al. [23] proposed hierarchical re-entry origami honeycomb (HROH), combining biomimetic hierarchical design with Miura origami patterns. Under low-, medium-, and high-speed impacts, the *SEA* of HROH increased by 36.37%, 13.36%, and 28.56%, respectively, compared to traditional structures. Shi et al. [24] designed a trapezoidal origami corrugated tube core acoustic metasurface, combining micro-perforated panels with an origami core. This structure exhibits excellent low-frequency sound absorption performance in the 480–620 Hz and 1120–1340 Hz frequency bands, while also outperforming traditional honeycomb and Miura core structures in energy absorption under low-speed impacts, achieving synergistic optimization of acoustic and mechanical performance.

Among these, biomimetic tree structures have been extensively studied by some scholars and have been proven to have better energy absorption effects. Inspired by the fractal structures of natural tree ladders, Chen et al. [25] designed a hexagonal hierarchical gradient structure (HHGS). Research indicates that impact resistance comparisons demonstrate that under inclined loading, the gradient structure significantly reduces initial peak forces compared to other structures while maintaining favorable deformation patterns and energy absorption advantages. Wang et al. [26] were inspired by the branching characteristics of lotus leaf veins to design a circular vein branching nested tube (CVBNT). The introduction of the vein branch thin-walled plates affects the deformation mechanism and leads to more plastic hinges, which will greatly improve the *SEA*. Ha et al. [27] mimicked the hierarchical structure of tree branches and lotus leaf veins to propose a biomimetic hierarchical multi-cell double tube (BHMB). The results demonstrate that the *SEA* increases with the hierarchical order and inner diameters. Ha et al. [28] investigated the impact resistance of a bio-inspired fractal multi-cell circular (BFMC). Its energy absorption rate (SER) was improved by 35.43% compared to conventional multi-unit tubular structures. The complex proportional assessment (COPRAS) method was adopted to optimize the performance of the BFMC. Wu et al. [29] proposed a tree-like fractal method for developing energy absorption structures. They explored triangular, quadrilateral, and pentagonal structures and found that the third-order tree-like fractal structure exhibited the best load-bearing capacity.

In summary, inspired by origami and tree branches, this study analyzes OTSs for collision energy absorption. Based on finite element models, multi-objective performance analysis and evaluation were conducted on OTSs with varying numbers of wave peaks. The OTS with optimal performance was selected as the target for subsequent optimization. The multi-objective optimization of the OTS was performed using the MOEA/D-DAE algorithm to obtain a set of Pareto optimal solutions. The optimal structure was then determined through the TOPSIS decision-making process. Subsequently, comparative analysis and discussion were performed to validate the effectiveness and practicality of this optimization approach.

Compared with previous studies, this paper summarizes two unique contributions: (1) A biomimetic thin-walled energy-absorbing structure integrating origami and tree-shaped structures was developed, and a subsequent analysis of its mechanical properties was conducted. OTSs offer significant advantages in reducing *IPCF* without substantially compromising other performance metrics. (2) A multi-criteria decision-making method integrating MOEA/D-DAE and TOPSIS has been developed. This integrated approach is employed to identify the optimal structure. This optimization framework enriches the methods in the field of optimization, providing an alternative optimization decision-making system.

The structure of this paper is as follows: Section 2 introduces the construction and validation of the OTS finite element (FE) model. Section 3 analyzes the influence of different OTS parameters on energy absorption performance. Section 4 employs a multi-criteria decision-making approach to identify the optimal structure. Finally, the conclusions of this paper are drawn in Section 5.

## 2. Structural Design and Simulation Model Construction of OTS

This section delineates the pertinent parameters for biomimetic prototyping and structural design. The validity of the finite element (FE) model was verified by comparing the results of the FE model with those of experiments. Furthermore, the model’s viability was validated by comparing the data indicators of the constructed model with those of a circular tube of equivalent dimensions.

### 2.1. Structural Design and Collision Design of OTS

**Figure 1:**
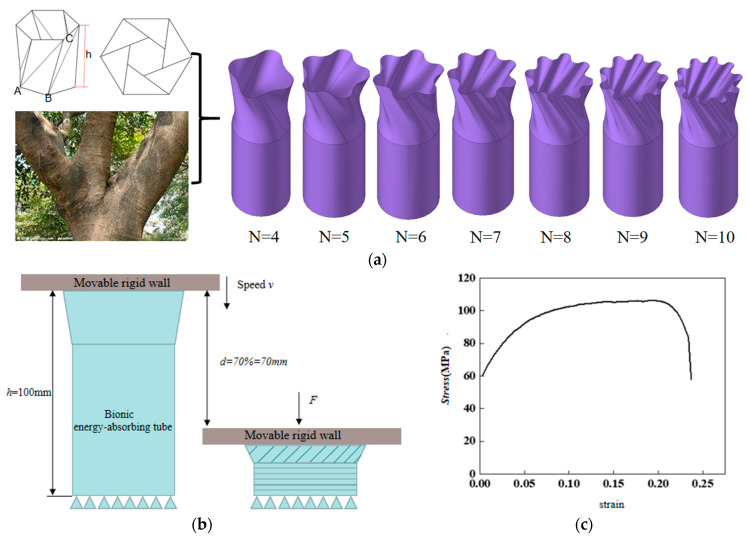
Structural design and simulation model construction of OTS. (**a**) Schematic diagram of OTS bionic prototype and OTS structure (*N* = 4–10). (**b**) Collision scheme in FEA: the rigid wall only moves at a certain speed along the direction of the Z axis and it stops at 70% of the height of the OTS. And the translation and rotation of the bottom of the OTS in the X, Y, and Z axis directions are completely restricted. (**c**) AA6061O effective stress–strain curve.

The problem under consideration involves an OTS, with its head serving as a buffer zone and the remaining parts composed of thin-walled circular tubes. The upper portion of the OTS is composed of tangent axis-symmetric arcs, resulting in the formation of an end face. In this study, the folds of the origami structure are transformed into surfaces. As illustrated in Figure 1a, the fundamental model of the structure under investigation is depicted. A series of structures can be obtained by determining four independent variables. These variables include the scanning line angle *θ*, the proportion *δ* of the OTS as the energy-absorbing head relative to the overall structure, the diameter *x* of the top base circle, and the number *N* of top arcs.

A finite element model was established based on the geometric shapes depicted in Figure 1a. The creation of models with peak numbers of 4, 5, 6, 7, 8, 9, and 10 is illustrated in Figure 1a. The parameter settings are as follows: the diameter of the top base circle, *x*, is 36 mm; the diameter of the bottom base circle, *y*, is 36 mm; the length of the energy absorption tube, *h*, is 100 mm; the head occupancy ratio, *δ*, is 0.4; the top wave peak height, *z*, is 3 mm; the scanning line angle, *θ*, is 60°; and the wall thickness, *t*, is 1 mm. The OTS grid size is 2 mm × 2 mm. The static friction coefficient is set to 0.3, and the dynamic friction coefficient is set to 0.28. The utilization of Belytschko–Tsay shell elements is a hallmark of the approach employed. A rigid wall is constrained and moves along the Z-axis at a speed of 1 mm/ms, representing a quasi-static condition. In order to guarantee that the OTS remains fixed when subjected to impact or compression from the rigid wall, the translation and rotation of the OTS bottom along the X, Y, and Z axes are fully constrained. The collision scheme is illustrated in Figure 1b.

The structural material is AA6061O [30]. The mechanical properties of AA6061O are *E* = 69 GPa, Poisson’s ratio ν = 0.3, and density ρ = 2700 kg/m^3^. Figure 1c shows the stress–strain curve for AA6061O.

### 2.2. Description of Crashworthiness Criteria

Let the final fragmentation displacement generated by the energy-absorbing tube be *d* [31,32]. Then, by integrating the *f-d* curve, the energy absorption (*EA*) is obtained as follows:

(1) $$EA\;=\int_{0}^{d}fdx,$$

To evaluate the energy absorption efficiency of the energy absorption tube, the *SEA* can be obtained from the ratio of the *EA* to the mass *m* of the energy absorption tube [33].

(2) $$SEA=\frac{EA}{m},$$

The *SEA* is the amount of energy that an energy-absorbing structure can absorb per unit mass, and it can more comprehensively reflect the strength of the structure’s *SEA*. The higher the specific *SEA*, the better the energy absorption effect of the structure.

To show the average level of contact force throughout the process, the ratio of the total energy EA absorbed by the energy-absorbing tube during the entire impact or compression process to the fracture displacement (*d*) can be used to obtain the average force *MCF* [33].

(3) $$MCF=\frac{EA}{d},$$

Since the contact force fluctuates during impact or compression, in order to evaluate the stability of the load on the energy-absorbing tube during impact, it is necessary to calculate the load fluctuation *ULC* [33].

(4) $$ULC=\frac{\int_{0}^{d}\left|F\left(x\right)\;-\;MCF\right|dx}{EA},$$

The three key indicators used in this study to evaluate the comprehensive performance of energy-absorbing tubes—*SEA*, *IPCF*, and undulation of the load-carrying capacity (*ULC*)—constitute the ultimate optimization objectives of this research.

### 2.3. FEM Feasibility Verification and Feasibility Verification

**Figure 2:**
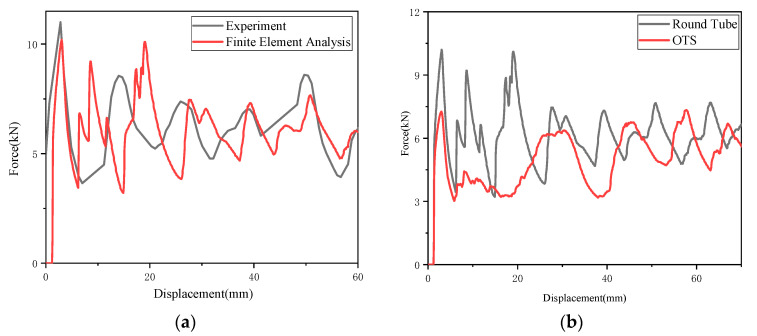

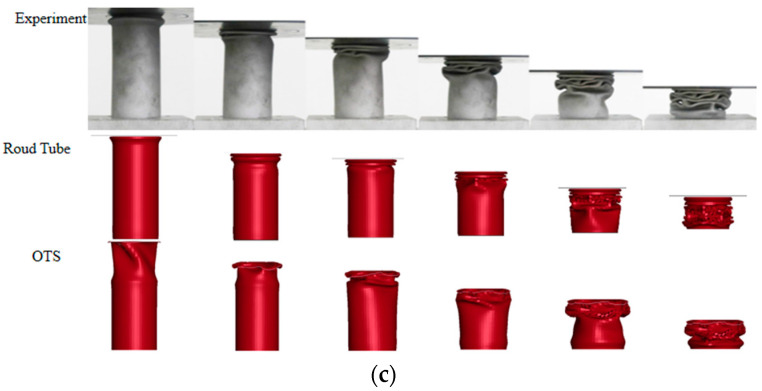
OTS FE model validation: (**a**) comparison of experimental and FEM *f-d* curves for round tubes; (**b**) comparison of *f-d* curves for round tubes and OTS; and (**c**) comparison of experimental and FEM deformation.

According to the experimental setup and results reported by Deng et al. [30], a circular pipe model of identical dimensions was constructed. As demonstrated in Figure 2c, the deformation patterns exhibited by the finite element model at varying compression displacements demonstrate a high degree of congruence with the experimental outcomes. Figure 2a presents the *f-d* comparison curves obtained from both simulation and experiment. The *IPCF* error was determined to be 7.576%, the *SEA* error was 0.693%, and the *ULC* error was 9.685%. The findings indicated that all errors fell within the 10% range, thereby validating the finite element model.

The fracture displacement was measured at 70 mm, and the comparison results with the circular pipe of equal cross-section are shown in Figure 2b. An analysis of Figure 2b reveals that the structure designed in this study effectively reduced *IPCF* by approximately 30%, thereby demonstrating exceptional *IPCF* attenuation capability. When subjected to compression, the front end of the OTS undergoes rotational deformation along the surface creases, exhibiting lower strength than conventional circular tubes and resulting in a reduced *IPCF*. Furthermore, the substantial decrease in *IPCF* did not excessively compromise the overall *SEA*, indicating that the model combines the advantages of both aspects, thereby validating the feasibility of the design.

## 3. Research on the Energy Absorption Characteristics of OTS

This section undertakes a rigorous analysis of the deformation modes of the OTS and determines the optimal number of wave peaks (*N*). Accordingly, a sensitivity analysis was conducted to examine the impact of various parameters (*θ*, *δ*, *x*) on energy absorption performance.

### 3.1. Effect of Number of Wave Peaks (N) on Crashworthiness of OTS

**Figure 3:**
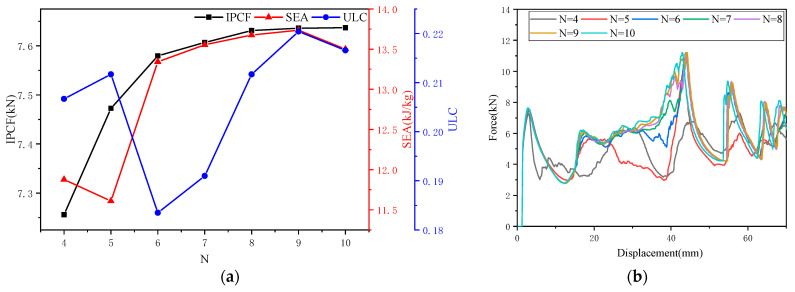

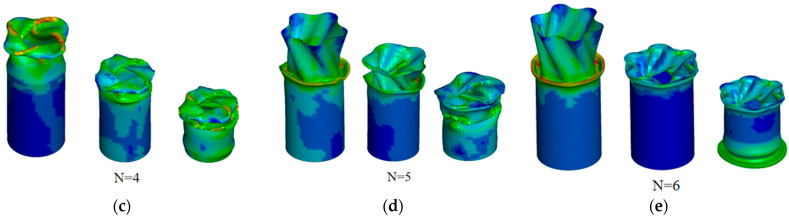
The effect of number of wave peaks (*N*) on crashworthiness: (**a**) the effect of *N* on three collision safety performance indicators; (**b**) *f-d* comparison curve of OTS (*N* = 4–10); (**c**) deformation pattern of OTS (*N* = 4) under quasi-static compression; (**d**) deformation pattern of OTS *(N* = 5) under quasi-static compression; and (**e**) deformation pattern of OTS (*N* = 6) under quasi-static compression.

As shown in Figure 3a, with increasing *N*, *IPCF* and *SEA* gradually increase, while *ULC* exhibits fluctuations. The corresponding *f-d* comparison curves for the OTS (*N* = 4–10) are shown in Figure 3b. As *N* increases, the force in the 20–40 mm range increases, and *EA* increases. The evaluation indicators were obtained based on data obtained from finite element simulation, and the results are shown in Table 1.

As the *N* value increases, the OTS exhibits three deformation patterns: In the OTS (*N* = 4), as shown in Figure 3c, the origami-inspired dendritic structure segment deforms first, followed by deformation of the circular tube segment. In the OTS (*N* = 5), the strength of the origami dendritic structure increases, and it begins to deform after partially embedding into the circular tube, with the circular tube deforming last (as shown in Figure 3d). In the OTS (*N* = 6), the origami tree structure further strengthens and becomes fully embedded within the circular tube. The bottom of the tube deforms first, followed by coordinated deformation of the embedded segment and the tube (as shown in Figure 3e). The deformation patterns for the OTS (*N* = 7–10) are similar to those of the OTS (*N* = 6).

Decision analysis was conducted using the TOPSIS [34,35] entropy weight method. The weight coefficient for *IPCF* was determined to be 57.57%, while *SEA* received a coefficient of 16.12%, and *ULC* was assigned a coefficient of 26.30%. It is evident that the weight coefficient for *IPCF* was considerably higher than those of the other two evaluation indicators. This finding is in alignment with the research objective of this paper, which is to reduce the *IPCF*. The findings suggest that the OTS (*N* = 4) demonstrates optimal energy absorption capabilities.

### 3.2. Effect of Different Parameters (θ, δ, x) on Crashworthiness of OTS

#### 3.2.1. Effect of *θ* on Crashworthiness of OTS

The scanning line angle (*θ*) is the object of study. The other parameters are set as follows: the top wave peak height is *z* = 3 mm, *δ* = 0.4, and *x* = 36 mm. The remaining parameters are consistent with those in the previous section. The range of settings for the scanning line angle, *θ*, is [60°, 120°], with an increment of 10° each time. Table 2 shows the energy absorption assessment under different scanning line angles. Through regression analysis, *θ* explains 97.5% of the variation in *IPCF*, 51.6% in *SEA*, and 83.7% in *ULC*.

**Figure 4:**
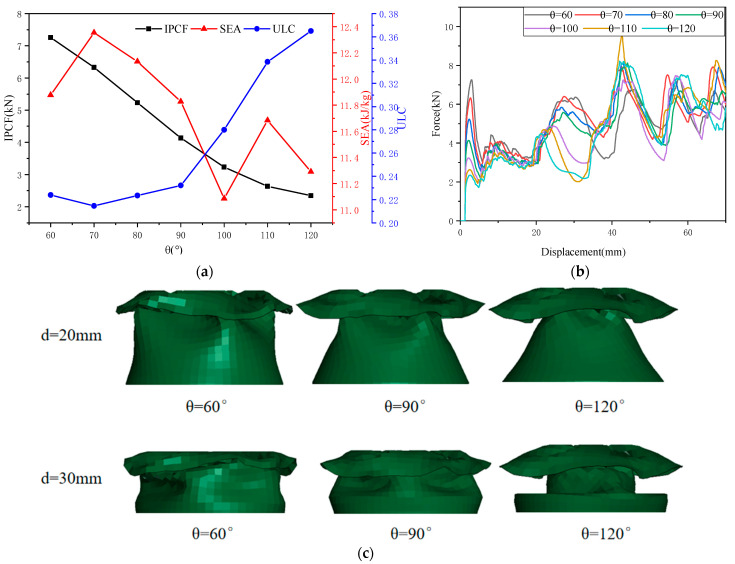
The effect of the scanning line angle (*θ*) on crashworthiness: (**a**) the effect of *θ* on three collision safety performance indicators; (**b**) *f-d* comparison curve of OTS (*θ* = 60–120°); and (**c**) deformation diagrams of OTS (*θ* = 60°, 90°, 120°) for *d* = 20 mm and 30 mm.

As shown in Figure 4a, as *θ* increases, the *IPCF* exhibits a monotonically decreasing trend, while the rate of change in *IPCF* shows a decreasing trend; as *θ* increases, the *SEA* exhibits a fluctuating trend and lacks monotonicity, reaching a minimum value at *θ* = 100° and a maximum value at *θ* = 70°; As *θ* increases, *ULC* first decreases and then increases, reaching a minimum at *θ* = 70° and a maximum at *θ* = 120°. As shown in Figure 4b, as *θ* increases, *IPCF* almost “disappears,” forming two gradually increasing platform force intervals (*d* = 0–20 mm) and (*d* = 40–70 mm); the energy absorption in the transition region (*d* = 20–40 mm) gradually decreases. As *θ* increases, the deformation pattern of the front section becomes progressively less folded, and *EA* gradually decreases, as shown in Figure 4c. Based on the data and trend line analysis, it can be concluded that the scanning line angle (*θ*) is a sensitive factor.

#### 3.2.2. Effect of *δ* on Crashworthiness of OTS

Taking the head occupancy ratio *δ* as the object of study, other parameters are set as *θ* = 60°, *x* = 36 mm, and other parameters are the same as in the previous section. The head occupancy ratio *δ* is set in the range [0.2, 0.5], with 0.1 as the unit of change. Different head occupancy ratio energy absorption evaluation indicators are shown in Table 3. Through regression analysis, *δ* explains 98.2% of the variation in *IPCF*, 26.3% in *SEA*, and 12.6% in *ULC*.

**Figure 5:**
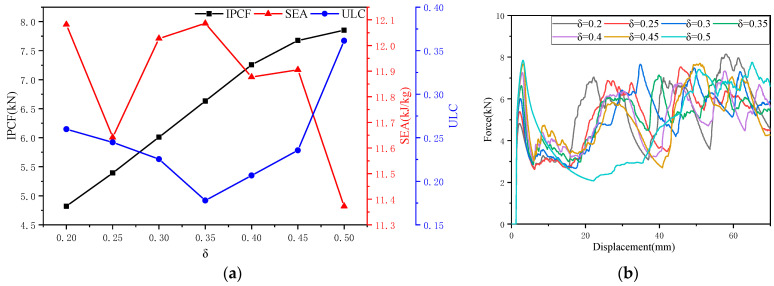

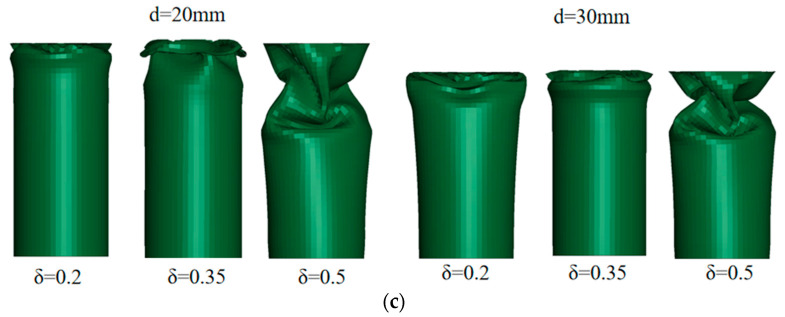
The effect of the head occupancy ratio (*δ*) on crashworthiness: (**a**) the effect of *δ* on three collision safety performance indicators; (**b**) *f-d* comparison curve of OTS (*δ* = 0.2–0.5); and (**c**) deformation diagrams of OTS (*δ* = 0.2, 0.35, 0.5) for *d* = 20 mm and 30 mm.

As shown in Figure 5a, as *δ* increases, *IPCF* monotonically increases; for *SEA*, the trend is fluctuating and lacks monotonicity, reaching a minimum at *δ* = 0.5 and a maximum at *δ* = 0.2; as *δ* increases, *ULC* first decreases and then increases, reaching a minimum at *δ* = 0.35 and a maximum at *δ* = 0.5. As shown in Figure 5b, as *δ* increases, the two-stage platform force gradually disappears; the energy absorption in the transition zone (*d* = 20–40 mm) gradually decreases, forming significant fluctuations. As *δ* increases, the deformation pattern remains relatively stable when *δ* < 0.45, but becomes unstable at *δ* = 0.5, consistent with the behavior of the *ULC*. See Figure 5c for details. Based on the data and trend line analysis, it can be concluded that head occupancy rate (*δ*) is a sensitive factor.

#### 3.2.3. Effect of *x* on Crashworthiness of OTS

The base circle diameter *x* of the top pattern was selected as the research object, and the other parameters were set as follows: *θ* = 90°, *δ* = 0.4, and the remaining parameters were consistent with the previous section. The value range of the base circle diameter *x* was set to [28 mm, 52 mm], with an increment of 4 mm. The energy absorption evaluation indicators for different head proportions are shown in Table 4. Through regression analysis, *x* explains 99.8% of the variation in *IPCF*, 96.8% in *SEA*, and 55.5% in *ULC*.

**Figure 6:**
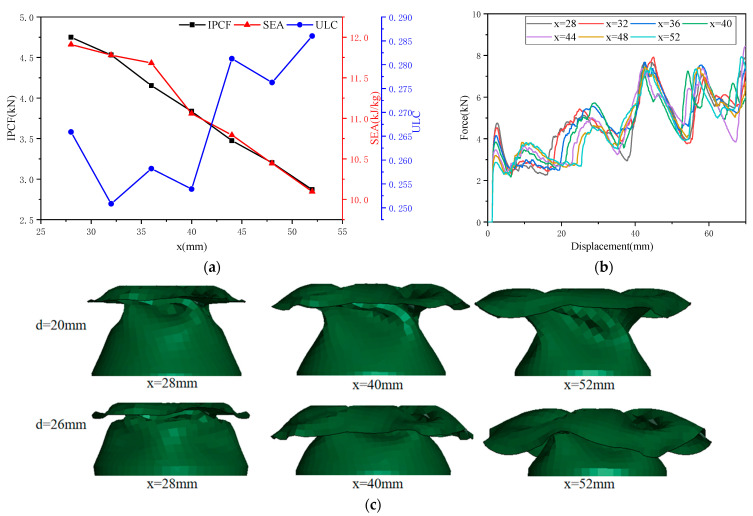
The effect of top base circle diameter (*x*) on crashworthiness: (**a**) the effect of *x* on three collision safety performance indicators; (**b**) *f-d* comparison curve of OTS (*x* = 28–52 mm); and (**c**) deformation diagrams of OTS (*x* = 28 mm, 40 mm, 52 mm) for *d* = 20 mm and 36 mm.

As shown in Figure 6a, as *x* increases, *IPCF* and *SEA* decrease monotonically; as *x* increases, *ULC* fluctuates but shows an overall upward trend. As shown in Figure 6b, as *x* increases, the first peak force gradually decreases, while the second peak force gradually increases; the energy absorption in the transition zone (*d* = 20–40 mm) gradually decreases; the overall *f-d* curves are similar; and *ULC* fluctuates. As *x* increases, the front-end deformation mode gradually becomes unstable, consistent with the behavior of *ULC*, as shown in Figure 6c. Based on the data and trend line analysis, it can be concluded that the top base circle diameter (*x*) is a sensitive factor.

## 4. Multi-Objective Optimization of OTS

Establishing an agent model enables faster identification of the optimal OTS [36]. The multi-criteria decision-making method integrates MOEA/D-DAE and TOPSIS. This integrated approach is employed to identify optimal structures. The efficacy of this approach is substantiated through a comparative analysis of the prediction outcomes with those derived from finite element analysis.

### 4.1. Multi-Objective Optimization Proxy Model Construction

**Figure 7:**
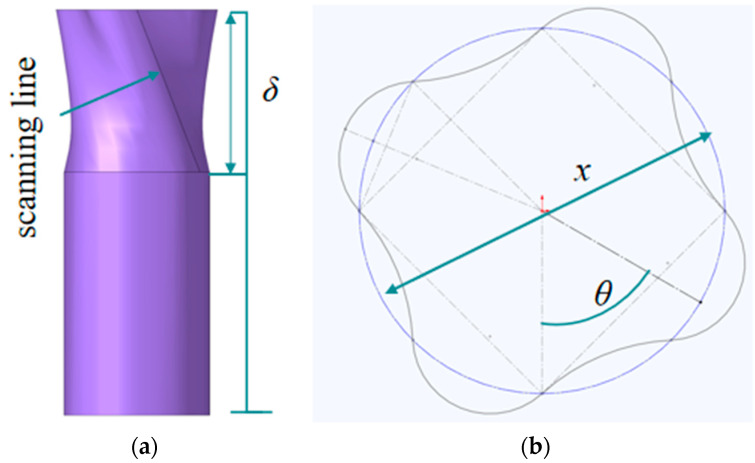
Optimized parameters *θ*, *δ*, and *x* of OTS. (**a**) Stereographic diagram of OTS; (**b**) cross-section of OTS.

The parameters *θ*, *δ*, and *x* of the OTS were selected as independent variables, as shown in Figure 7. Their respective ranges are *δ* = 0.2–0.5, *x* = 28–52 mm, and *θ* = 60–120°. The three optimization objectives selected for this study are *IPCF*, *SEA*, and *ULC*.

**Figure 8:**
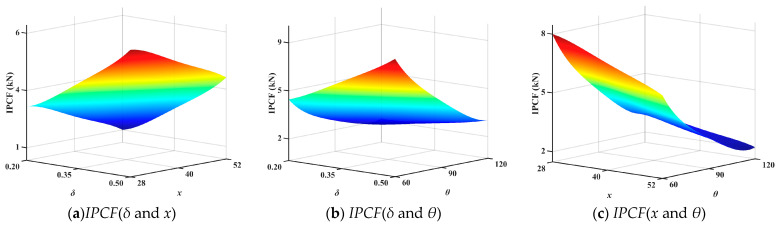
Second-order interaction response surface of *IPCF*.

**Figure 9:**
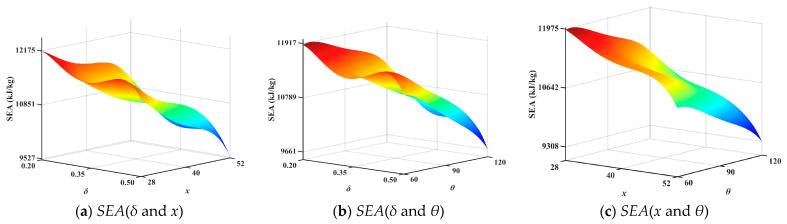
Second-order interaction response surface of *SEA*.

**Figure 10:**
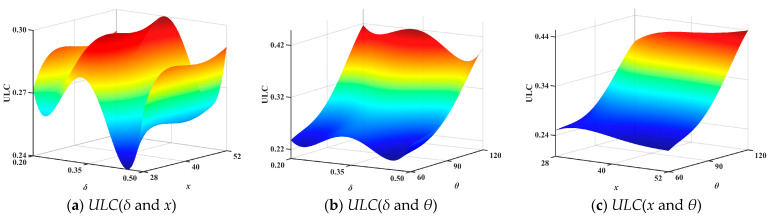
Second-order interaction response surface of *ULC*.

The optimal Latin hypercube sampling method was used to obtain 32 sets of numerical samples. Establish 32 sample models for FEA. Obtain *IPCF*, *SEA*, and *ULC* to construct the surrogate model. The response surfaces showing the interactions between the variables (*θ*, *δ*, *x*) and the optimization objectives (*IPCF*, *SEA* and *ULC*) are shown in Figure 8, Figure 9 and Figure 10. To ensure the accuracy of the surrogate model, error analysis is typically performed. Typical error assessment metrics for surrogate models include maximum value (*MAX)*, root mean square error (*RMSE*), mean relative error (*MRE*), and coefficient of determination (*R*^2^). RMSE indicates the global accuracy of the surrogate model; lower values indicate higher accuracy, typically not exceeding 0.2. MRE indicates the local accuracy of the surrogate model; lower values indicate higher accuracy. A higher R^2^ value indicates higher accuracy, with values approaching 1. The errors of the established surrogate model are calculated in this paper, as shown in Table 5.

### 4.2. Multi-Objective Parameter Optimization of OTS

**Figure 11:**
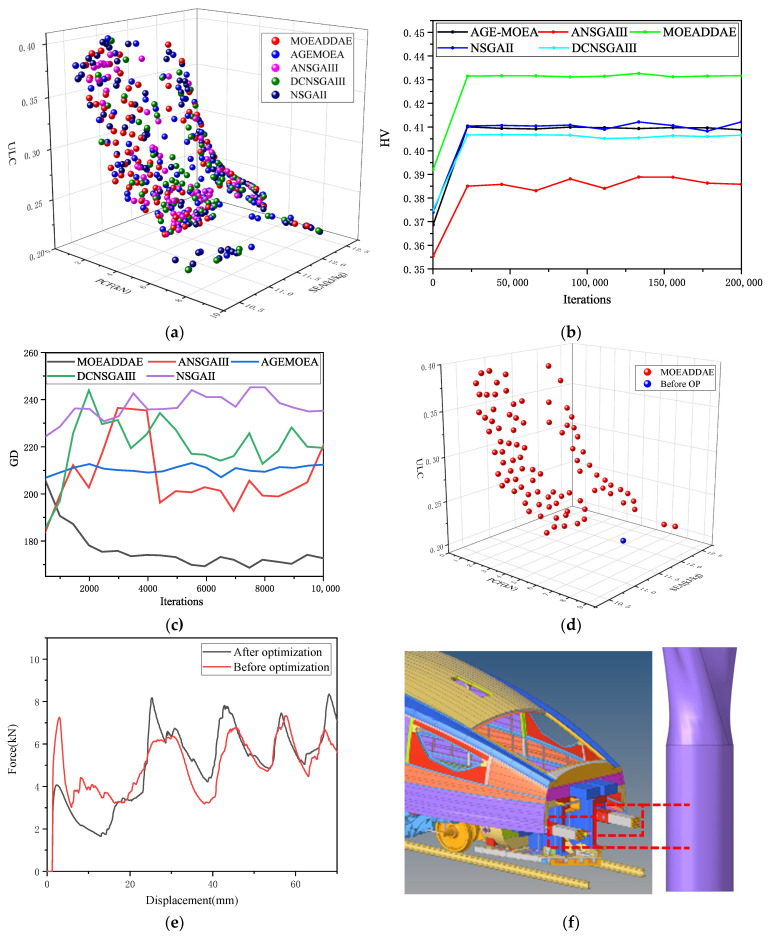
Multi-objective parameter optimization of OTS: (**a**) Pareto solution sets of each algorithm in the spatial diagram; (**b**) *HV* curves of each algorithm; (**c**) *GD* curves of each algorithm; (**d**) optimization results of the MOEA/D-DAE algorithm; (**e**) comparison curves of *f-d* before and after optimization; and (**f**) application of OTS in the primary energy absorption structure of rail vehicles.

Five optimization algorithms MOEA/D-DAE [37], AGE-MOEA [38], DCNSGAIII [39], NSGAII [40,41], and ANSGAIII [42]—were employed for comparative analysis. These optimization methods were applied under the following constraints: 60° ≤ *θ* ≤ 120°, 0.2 ≤ *δ* ≤ 0.5 and 28 mm ≤ *x* ≤ 52 mm. *SEA* is a profitability indicator, with higher values being better; *IPCF* and *ULC* are cost indicators, with lower values being better. The smaller the values of PCF and *ULC* are and the higher the value of *SEA* is, the better the performance of the OTS. Thus, the mathematical theoretical model of optimal design can be established as shown in Equation (5). The target values corresponding to the Pareto solutions are marked in the three-dimensional diagram to illustrate their relationships, as shown in Figure 11a.

(5) $$\begin{array}{c} Maxmize[SEA\left(\theta ,\delta ,x\right)]\\ Minimize[IPCF\left(\theta ,\delta ,x\right)]\\ Minimize[ULC\left(\theta ,\delta ,x\right)]\\ 60{}^{\circ}<\theta <120{}^{\circ}\\ 0.2<\delta <0.5\;\\ 28\;\mathrm{mm}<x<52\;\mathrm{mm} \end{array},$$

Based on the shape of the Pareto front, it is impossible to determine whether the optimization results of an algorithm are good or bad. Therefore, we introduce the hypervolume *HV* [43] and generational distance *GD* [44] as evaluation metrics for algorithm performance to assess the quality of optimization results for each algorithm.

*HV* is the volume of the region enclosed by the non-dominated solution set obtained by the algorithm and the reference point in the target space. Its formula is as follows:

(6) $$HV\;=\;\delta ({⋃}_{i=1}^{\;|S|}v_{i}),$$

Here, *δ* denotes the Lebesgue measure, which is used for volume measurement; |*S*| denotes the number of non-dominated solution sets; and $v_{i}$ denotes the hypervolume formed by the reference point and the *i*-th solution in the solution set.

*HV* can simultaneously evaluate the convergence and diversity of an algorithm population. The larger the *HV* value of an optimization algorithm, the better its overall performance. Figure 11b shows the *HV* curves for each algorithm.

Figure 11b shows the line chart of the *HV* values for each algorithm. It can be seen that the *HV* value of the MOEA/D-DAE algorithm is the highest, fluctuating around 0.43 and tending to stabilize. For the AGE-MOEA algorithm, NSGA-II algorithm, and DCNSGA-III algorithm, their *HV* values are all below 0.42. The ANSGA-III algorithm performs the worst, with *HV* values below 0.39 and significant fluctuations. The MOEA/D-DAE algorithm demonstrates higher *HV* values than the other four algorithms at all stages of iteration, and its line chart is also more stable.

*GD* measures the quality of convergence from the approximate set *A* to the Pareto frontier (*PF*). The formula is as follows:

(7) $$GD\;=\;\frac{1}{\left|A\right|}{\left(\sum_{i=1}^{\left|A\right|}{dis(a_{i},PF^{\prime })}^{p}\right)}^{\frac{1}{p}},$$

Here, *PF′* is a subset of *PF* and $dis(a_{i},PF^{\prime })$ is the distance from $a_{i}$ to *PF′*.

*GD* measures the average distance between algorithm solutions and the *PF*. The smaller the *GD* value of an optimization algorithm, the better its convergence. Figure 11c displays the *GD* curves for each algorithm.

Figure 11c shows the line chart of the *GD* values for each algorithm. Overall, the MOEA/D-DAE algorithm demonstrated the best convergence performance, exhibiting a sustained downward trend throughout the process with a more pronounced decrease in the later stages, ultimately achieving a low *GD* value. This indicates that during iteration, the generated approximate solution set continuously approached the true Pareto frontier, with convergence performance steadily improving as iterations progressed.

Figure 11d compares the optimized Pareto frontier surface with the pre-optimization data. The optimized *IPCF* values are almost entirely lower than the pre-optimization values. The optimized *SEA* values are partially higher than the pre-optimization values, while the optimized *ULC* values are mostly higher than the pre-optimization values, with only a small portion slightly lower than the pre-optimization values.

One of the optimized results, the OTS (*θ* = 102.05°, *δ* = 0.214, *x* = 34.57 mm), was used to construct a finite element model. The error between the finite element results and the surrogate model is shown in Table 6. Compared with the evaluation index of the structure before optimization, the *IPCF* was reduced by 70.8%.

Then, the TOPSIS entropy weight method was used for decision-making, where $W_{SEA}$ = 53.43%, $W_{IPCF}$ = 8.86%, and $W_{ULC}$= 37.71%. The optimal parameters OTS were determined as *θ* = 65.84°, *δ* = 0.225, and *x* = 28 mm. Its *IPCF* is 4.9919 kN, *SEA* is 12.316 kJ/kg, and *ULC* is 0.2282. Compared to the evaluation metrics of the pre-optimized structure, *IPCF* decreased by 31.2041%, *SEA* increased by 3.5647%, and *ULC* increased by 10.4265%. The comparison curve of *f-d* before and after optimization is shown in Figure 11e.

## 5. Conclusions

This study proposes the design and analysis of an OTS. First, the geometric concepts and parameters of OTS are outlined. Subsequently, by comparing finite element analysis (FEA) with experimental results, it is found that the force–displacement curves obtained from experiments and FEA are basically consistent, further validating the effectiveness of the finite element model. The entropy-weighted TOPSIS comprehensive evaluation method is employed for multi-attribute decision analysis, identifying OTS (*N* = 4) as the optimal design scheme. The effects of different *N*, *θ*, *δ*, and *x* on the OTS’s *IPCF*, *SEA*, and *ULC* are investigated, as detailed in Table 7.

Subsequently, five optimization algorithms—MOEA/D-DAE, AGE-MOEA, ANSGAIII, NSGAII, and DCNSGAIII—were compared. Compared to other algorithms, the MOEA/D-DAE algorithm demonstrated the highest hypervolume (*HV*) value and the smallest generational distance (*GD*) value, indicating its superior suitability for this engineering problem. The effectiveness of the agent model was validated by comparing finite element analysis (FEA) results with the predicted results. Finally, the TOPSIS comprehensive evaluation method was used to adjust the objective weights to obtain the optimal solution. Compared to the OTS before optimization, the results showed that the *IPCF* was reduced by 31.2%, the *SEA* increased by 3.6%, and the *ULC* increased by 10.4%. OTSs can significantly reduce *IPCF* without substantially compromising other metrics. It can thus be seen that OTSs offer new insights for the future design of thin-walled energy-absorbing structures. OTSs can be applied to the primary energy-absorbing structures of rail vehicles (as shown in Figure 11f), as well as to other applications such as crash cushions required for highway maintenance tasks.

In future studies, we will focus on two directions: (1) investigating whether bottom cross-sections of different shapes can enhance OTS performance, and (2) by combining multiple OTSs, we investigate the effects of OTS quantity, surface geometry, head coverage ratio, and OTS group distribution on overall performance to achieve self-cancelation effects through interleaved superposition of *ULC* at different displacements.

## Figures and Tables

**Table 1:** Energy absorption evaluation index of OTS (*N* = 4–10).

*N*	*IPCF*/kN	*SEA*/(kJ/kg)	*ULC*
4	7.2561	11.8770	0.2067
5	7.4726	11.6097	0.2117
6	7.5797	13.3434	0.1835
7	7.6069	13.5550	0.1910
8	7.6311	13.6766	0.2117
9	7.6359	13.7357	0.2204
10	7.6367	13.5014	0.2166

**Table 2:** Energy absorption evaluation index of OTS (*θ* = 60–120°).

*θ*/°	*IPCF*/kN	*SEA*/(kJ/kg)	*ULC*
60	7.2561	11.8770	0.2239
70	6.3277	12.3541	0.2146
80	5.2330	12.1334	0.2235
90	4.1355	11.8280	0.2322
100	3.2313	11.0869	0.2800
110	2.6370	11.6841	0.3386
120	2.3448	11.2910	0.3650

**Table 3:** Energy absorption evaluation index of OTS (*δ* = 0.2–0.5).

*δ*	*IPCF*/kN	*SEA*/(kJ/kg)	*ULC*
0.2	4.8203	12.0821	0.2598
0.25	5.3925	11.6416	0.2447
0.3	6.0099	12.0275	0.2255
0.35	6.6303	12.0866	0.1779
0.4	7.2561	11.8770	0.2067
0.45	7.6755	11.9056	0.2355
0.5	7.8512	11.3726	0.3613

**Table 4:** Energy absorption evaluation index of OTS (*x* = 28–52 mm).

*x*/mm	*IPCF*/kN	*SEA*/(kJ/kg)	*ULC*
28	4.749715	11.91138	0.26588
32	4.531363	11.7802	0.250811
36	4.154067	11.68159	0.258188
40	3.835862	11.05811	0.253902
44	3.474882	10.79251	0.281252
48	3.203085	10.44484	0.276268
52	2.870313	10.09592	0.285989

**Table 5:** Proxy model error value.

	*MAX*	*RMSE*	*MRE*	*R* ^2^
Acceptance	0.3	0.1	0.1	0.9
*IPCF*	0.05088	0.02114	0.01788	0.99462
*SEA*	0.16272	0.06662	0.05533	0.93062
*ULC*	0.21789	0.08413	0.06623	0.90783

**Table 6:** Comparison between the agent model and FEA model.

	*IPCF*/kN	*SEA*/(kJ/kg)	*ULC*
Agent model	2.12	11.57	0.329
FEA	2.18	11.61	0.325
Error	2.80%	0.33%	1.14%

**Table 7:** Effects of OTS Key Parameters on Mechanical Performance.

OTS Key Parameter	Mechanical Performance Changes
*N*	As the *N* of the OTS increases, the peak force in the transition zone grows larger, exhibiting three distinct deformation patterns.
*θ*	As the *θ* of the OTS increases, the *IPCF* and front-end peak force decrease, even disappearing entirely, forming two progressively increasing plateau force intervals. Energy absorption in the transition zone gradually diminishes.
*δ*	As the *δ* of the OTS increases, the two-stage plateau forces gradually disappear; energy absorption in the transition zone diminishes progressively, forming distinct fluctuations.
*x*	As the *x* of the OTS increases, the first peak force gradually decreases while the second peak force gradually increases; energy absorption in the transition zone diminishes progressively, with overall *f-d* curves exhibiting similarity.

## Data Availability

The datasets used and/or analyzed during the current study are available from the corresponding author on reasonable request. All data generated or analyzed during this study are included in this article.
